# Supplementary material for: Common permutation methods in animal social network analysis do not control for non-independence
Source: Behav Ecol Sociobiol. 2022 Oct 29;76(11):151. doi: 10.1007/s00265-022-03254-x (PMC9617964; doi:10.1007/s00265-022-03254-x)
Supplement: Supplementary file 1 — Supplementary file1 (DOCX 27 KB) [file 265_2022_3254_MOESM1_ESM.docx]

**Supplementary Material: Common Permutation Methods in Animal Social Network Analysis Do Not Control for Non-independence**

Jordan D. A. Hart^1^, Michael N. Weiss^1,2^, Lauren J. N. Brent^^[[1]](#footnote-1)^1^, and Daniel W. Franks^*3^

^1^Centre for Research in Animal Behaviour, University of Exeter, UK

^2^Center for Whale Research, Friday Harbour, WA, USA

^3^Departments of Biology and Computer Science, University of York, UK

**Correspondence:** jordan.da.hart@gmail.com

**Running title:** Permutations and Non-independence

**Author contributions:** JDAH conceived the idea of the manuscript. The arguments were developed and refined by JDAH, MNW, LJNB, and DWF. The simulations were developed by JDAH with input from MNW, LJNB, and DWF. The manuscript the written by JDAH with input from MNW, LJNB, and DWF.

**Data availability statement:** The R code required to repeat the simulations has been deposited at: [https://doi.org/10.5281/zenodo.4903396.](https://doi.org/10.5281/zenodo.4903396)

**Keywords:** Animal social network analysis, mixed models, node-label permutations, permutation tests

# Supplementary Material: Examples for Multimembership Models in brms and MCMCglmm

This notebook is a short example to demonstrate how multimembership models can be used in place of QAP in dyadic regression. This can be done in a few different R packages, here we show how it can be done in both brms and MCMCglmm.

## Loading packages

Load the packages. asnipe is included to demonstrate the methods against MRQAP using the double semi-partialling method (Dekker et al. 2007).

library(asnipe) library(MCMCglmm) library(brms)

## Simulate dataset

Next we simulate a dataset with 20 nodes where edge weight (association strength) depends on the sexes of pairs of nodes and the age difference between the nodes. Some individuals are also just more likely to form links, and this creates a node dependence. In this imagined scenario, edge weights are more likely between individuals of different sexes with a large age difference.

set.seed(1)

# Simulate dyadic data with nodal dependence.

num_nodes <- 20

node_dependence <- rnorm(num_nodes) sexes <- sample(c(1, 2), num_nodes, replace=TRUE) mean_ages <- c(20, 10) ages <- rpois(num_nodes, mean_ages[sexes])

sex_diff <- abs(matrix(rep(sexes, num_nodes), num_nodes, num_nodes)

- t(matrix(rep(sexes, num_nodes), num_nodes, num_nodes))) age_diff <- abs(matrix(rep(ages, num_nodes), num_nodes, num_nodes)
- t(matrix(rep(ages, num_nodes), num_nodes, num_nodes))) dependencies <- matrix(rep(node_dependence, num_nodes), num_nodes, num_nodes) dependencies <- dependencies + t(dependencies)

error <- matrix(rnorm(num_nodes^2), num_nodes, num_nodes) error <- error + t(error) edge <- 0.2 * sex_diff + 0.2 * age_diff + dependencies + error

Y <- edge

X_age <- age_diff

X_sex <- sex_diff

We now have three matrices: Y holds edge weights, X_age holds age differences between pairs, and X_sex holds sex differences (binary) between pairs. This is the format required to conduct standard QAP/MRQAP.

## MRQAP

Use asnipe to demonstrate a dyadic regression analysis using conventional MRQAP:

mrqap.dsp(Y ~ X_age + X_sex)

### Output ###

MRQAP with Double-Semi-Partialing (DSP)

Formula: Y ~ X_age + X_sex

Coefficients:

Estimate P(b>=r) P(b<=r) P(|b|<=|r|)

intercept 0.77093807 0.993 0.007 0.007

X_age 0.07410393 0.980 0.020 0.055

X_sex 1.06371688 1.000 0.000 0.000

Residual standard error: 1.784 on 187 degrees of freedom

F-statistic: 18.97 on 2 and 187 degrees of freedom, p-value: 3.157e-08

Multiple R-squared: 0.1687 Adjusted R-squared: 0.1598

AIC: -168.7332

This tells us that age difference is nearly significant and that sex difference is significant.

## Prepare dataframe for brms and MCMCglmm

Now to apply the multimembership model using brms and MCMCglmm. Firstly, both of these packages expect a dataframe, so we need to encourage the data into the correct format. Because our network is undirected, this can be done by taking the upper triangle of each dataframe. We also need to generate a list of node IDs that correspond to the nodes of the network. This is how we will capture the multimembership aspect of the model.

**Note** If working with directed networks, the lower triangle will also need to be included and additional random effects may need to be included to account for influence of a node being a sender or a receiver. These decisions will depend on the data and question, and need to be carefully considered.

num_nodes <- dim(Y)[1]

node_ids_i <- matrix(rep(1:num_nodes, num_nodes), num_nodes, num_nodes) node_ids_j <- t(node_ids_i)

df <- data.frame(

edge_weight=Y[upper.tri(Y)], age_difference=X_age[upper.tri(X_age)], sex_difference=X_sex[upper.tri(X_sex)], node_id_1=factor(node_ids_i[upper.tri(node_ids_i)], levels=1:num_nodes), node_id_2=factor(node_ids_j[upper.tri(node_ids_j)], levels=1:num_nodes)

) head(df)

### Output ###

edge_weight age_difference sex_difference node_id_1 node_id_2

1 0.3831433 5 0 1 2

2 -0.4928128 7 1 1 3

3 3.6452428 12 1 2 3

4 2.6145456 5 0 1 4

5 0.2425129 0 0 2 4

6 4.1312292 12 1 3 4

Now we can look at applying multimembership models using MCMCglmm and brms.

## Multimembership models in MCMCglmm

Let’s start with MCMCglmm. The fixed effects part of the model is standard. To include the multimembership part, we use random effects and the mm function with the following notation:

fit_mcmc <- MCMCglmm(edge_weight ~ age_difference + sex_difference, random=~mm(node_id_1 + node_id_2), data=df)

summary(fit_mcmc)

### Selected Output ###

post.mean l-95% CI u-95% CI eff.samp pMCMC

(Intercept) 0.52375 -0.47178 1.45362 1000 0.292

age_difference 0.13727 0.06491 0.21220 1000 <0.001 ***

sex_difference 0.73020 0.12916 1.28318 1000 0.018 *

---

Signif. codes: 0 ‘***’ 0.001 ‘**’ 0.01 ‘*’ 0.05 ‘.’ 0.1 ‘ ’ 1

## Multimembership models in brms

brms uses a more conventional notation similar to lme4. Again, the function mm is used to include the multimembership random effects. Note that in this function, the effects are included as separate arguments, separated by a comma, instead of a sum notation like in MCMCglmm.

fit_brm <- brm(edge_weight ~ age_difference + sex_difference

+ (1 | mm(node_id_1, node_id_2)), df) summary(fit_brm)

### Output ###

Group-Level Effects:

~mmnode_id_1node_id_2 (Number of levels: 20)

Estimate Est.Error l-95% CI u-95% CI Rhat

sd(Intercept) 1.89 0.36 1.31 2.71 1.01

Population-Level Effects:

Estimate Est.Error l-95% CI u-95% CI Rhat

Intercept 0.54 0.47 -0.39 1.49 1.01

age_difference 0.14 0.04 0.06 0.22 1.00

sex_difference 0.72 0.29 0.15 1.30 1.00

In both of these multimembership models, we see that the coefficient estimates are quite different to those from QAP and have interpretable confidence/credible intervals. This is because these models account for confounds when calculating both the significance (where applicable) and in the effect size estimates, whereas QAP only accounts for confounds when calculating the significance.

1. Joint senior authors. [↑](#footnote-ref-1)
